# Sex differences in behavioral decision-making and the modulation of shared neural circuits

**DOI:** 10.1186/2042-6410-3-8

**Published:** 2012-03-21

**Authors:** William R Mowrey, Douglas S Portman

**Affiliations:** 1 Center for Neural Development and Disease, School of Medicine and Dentistry, University of Rochester, 601 Elmwood Avenue, Box 645, Rochester, NY 14642, USA; 2 Department of Biomedical Genetics, School of Medicine and Dentistry, University of Rochester, 601 Elmwood Avenue, Box 645, Rochester, NY 14642, USA; 3 Department of Biology, University of Rochester, 601 Elmwood Avenue, Box 645, Rochester, NY 14642, USA; 4 Janelia Farm Research Campus, 19700 Helix Drive, Ashburn, VA 20147, USA

**Keywords:** Sex differences, Neuromodulation, Decision-making, Invertebrates, Neuroscience, Neural circuits, Neuroethology

## Abstract

Animals prioritize behaviors according to their physiological needs and reproductive goals, selecting a single behavioral strategy from a repertoire of possible responses to any given stimulus. Biological sex influences this decision-making process in significant ways, differentiating the responses animals choose when faced with stimuli ranging from food to conspecifics. We review here recent work in invertebrate models, including *C. elegans*, *Drosophila*, and a variety of insects, mollusks and crustaceans, that has begun to offer intriguing insights into the neural mechanisms underlying the sexual modulation of behavioral decision-making. These findings show that an animal's sex can modulate neural function in surprisingly diverse ways, much like internal physiological variables such as hunger or thirst. In the context of homeostatic behaviors such as feeding, an animal's sex and nutritional status may converge on a common physiological mechanism, the functional modulation of shared sensory circuitry, to influence decision-making. Similarly, considerable evidence suggests that decisions on whether to mate or fight with conspecifics are also mediated through sex-specific neuromodulatory control of nominally shared neural circuits. This work offers a new perspective on how sex differences in behavior emerge, in which the regulated function of shared neural circuitry plays a crucial role. Emerging evidence from vertebrates indicates that this paradigm is likely to extend to more complex nervous systems as well. As men and women differ in their susceptibility to a variety of neuropsychiatric disorders affecting shared behaviors, these findings may ultimately have important implications for human health.

## Review

### Introduction: Biological sex as one dimension of internal state

Animal behavior is flexible. From moment to moment, a given sensory stimulus can elicit qualitatively different behavioral responses. Novel objects may be approached or avoided, food items may be pursued or ignored, conspecifics may be courted or attacked. Moreover, animals presented with multiple stimuli generally select a single behavioral strategy from a repertoire of possible responses. This behavioral flexibility is born, at least in part, out of necessity. Most complex behaviors engage the body of the animal as a whole, and thus are expressed in a mutually exclusive manner. This enforces a decision-making process, through which behaviors are prioritized according to the current physiological needs and reproductive goals of the animal [1,2]. Thus, animals do not behave as automatons. Rather, the mapping of sensory stimuli to motor output is flexible, and responsive to changes in their "internal state" (a rubric representing the synthesis of physiological needs and motivational drives). If an animal is malnourished, it will vigorously pursue food-related stimuli; if well-fed, it may ignore them and save valuable energy or avoid the risk of predation. In this way, behavioral flexibility makes a critical contribution to an animal's survival and reproductive success.

As animals reach sexual maturity, new dimensions are added to this calculus of internal state. In addition to growth and survival, the organism's behavioral decision-making process must now incorporate drives to locate and select mates, foster progeny, and compete for territory. This transition to reproductive maturity can differentially affect the sexes' behavioral decision-making in several important ways. The most prominent of these changes is the emergence of new behaviors that are sex-biased or sex-limited in their expression. These include many behaviors closely connected to reproduction, such as mating, courtship, offspring care, and aggressive behaviors. Notably, both sexes often retain the capacity to express these behaviors [3], though they are nonetheless expressed with greater frequency or in different contexts in the two sexes. A second related change is the differential prioritization of behaviors. It is not uncommon that behavioral programs expressed by both sexes (*e.g.*, feeding) are subordinated to those more directly related to reproduction (*e.g.*, mating or offspring care) by adult animals in certain contexts. As the expression of both shared and reproductive behaviors are linked through the decision-making process, reproductive behaviors are often associated with differences in the regulation of behaviors common to both sexes. Finally, behavioral priorities may differ between the two sexes even within the context of a single shared behavior. For example, preference for specific food items or food-related odors may differ significantly between the sexes. Such changes in shared homeostatic behaviors, such as feeding, drinking, and sleep, are often commensurate with the different metabolic demands of reproduction in the two sexes. Thus, behavioral decision-making is highly specialized in each sex, deeply affecting the expression of both sex-specific and shared behaviors.

How sex differences in behavioral decision-making emerge remains an important open question. Work in many systems has shown that a class of neurotransmitters known as neuromodulators have key roles in shaping behavioral prioritization. These molecules include monoamine neurotransmitters (such as dopamine, serotonin, and norepinephrine), acetylcholine, and a broad array of peptide transmitters. While some of these molecules can act as classical transmitters, neuromodulators are distinguished by their ability to modulate the physiological properties of neurons on comparatively long time scales [4]. These modulatory actions can take distinct forms in different cell types, and can alter a cell's spontaneous activity or response to input in diverse ways. Such disparate actions are often coordinated broadly throughout neural circuits, or even the entire nervous system, through the broad release of these substances in response to salient environmental stimuli or changes in physiological state [5]. Their ability to globally alter the function of the nervous system has implicated neuromodulators in the regulation of arousal, mood, and, more generally, behavioral state. These actions enable neuromodulators to alter the mapping of sensory input to behavioral output in response to changing internal state parameters, such as feeding status [6], stress [7], and circadian cycle [8], thus implementing changes in behavioral prioritization. An emerging literature is now drawing important links between neuromodulatory systems and sex differences in behavioral decision-making [9]. Many of these new insights have come from simpler animal models, particularly invertebrate species that offer superior genetic manipulability (*Drosophila *and *C. elegans*), physiological access (crustacean and molluscan systems), or behavioral models (insects and others). These studies have provided numerous examples in which modulated circuit function underlies the expression of behaviors driven by physiological status (feeding) and innate drives (aggression and courtship). In particular, the sex-dependent functional modulation of neural circuits common to both males and females is emerging as a key aspect of the neural basis for sex differences in behavior. Where attempts to explain behavioral sex differences in terms of the necessity or sufficiency of sex-specific neural structures have often been frustrated (as detailed in [3]), these findings point toward a new paradigm wherein nominally shared neural structures can form the substrates of these differences.

Significantly, sex differences in shared aspects of behavior are not limited to simple animals, but are also extensively documented in vertebrates, both in the lab and in the wild, and in human psychology. In humans, significant sex differences have been found in olfactory ability [10], thermoregulation [11], aggression [12], and other aspects of behavior [13-15]. Though many of these differences may be influenced by psychosocial factors [16,17], animal studies indicate that at least some sex differences in human behavior are likely to have biological underpinnings [18]. Notably, humans also exhibit significant sex-bias in the incidence of neuropsychiatric disorders affecting shared behaviors, including autism, ADHD, schizophrenia, depression, and anorexia [19,20]. The idea that biological underpinnings of sex differences in shared behaviors might contribute to this bias is intriguing, and suggests a better understanding of these issues could have a significant impact on human health. Interestingly, neuromodulatory systems, in addition to their important roles in implementing behavioral flexibility, have also been implicated in the etiology of a wide variety of mental health disorders exhibiting sex bias [21-24]. Investigating the roles of neuromodulatory systems in regulating sex differences in shared behaviors could thus provide important insight into both the mechanisms by which modulated circuit function alters behavior and the bases for disease susceptibility in man.

In the following sections, we review classical and recent findings that link the modulation of shared neural circuit function to sex differences in behavioral decision-making. Studies of behavioral prioritization (*e.g.*, feeding *vs*. copulation), feeding preference, motor behavior and nominally sex-specific behaviors (*e.g.*, aggression, courtship) all highlight the notion that biological sex intersects with other dimensions of an animal's internal state—*e.g.*, nutritional and reproductive status—to adaptively reshape the decision-making process. In several cases it can be shown that this sexual modulation of behavior emerges through dynamic functional alterations to the properties of shared neural circuits.

### Sex differences in behavioral prioritization: Modulated function of shared circuitry mediates competition between shared and sex-specific behaviors

As animals reach sexual maturity, the demands of reproduction introduce new constraints on their behavior. Motivation for feeding must be balanced not only with homeostatic drives for sleep, thermoregulation, and drinking, but also with drives for mating and fostering offspring. Importantly, this shifting of behavioral priorities upon reproductive maturation can affect the expression of shared behaviors in dramatic ways. For example, drives to mate or to care for offspring can sometimes supersede drives to seek food [25], avoid predation [26], or even breathe [1]! The mechanisms underlying such changes in behavioral priorities are not well understood. However, a number of peptide and monoamine neuromodulators have been found to play critical roles in the regulating the motivation for specific behaviors as a function of an animal's physiology [27,28]. Evidence emerging from invertebrate models has offered new perspectives on how these signaling systems can influence behavioral prioritization and decision-making when sex-specific and reproductive behaviors compete for expression.

It has long been recognized in many species that sexual behaviors exhibit homeostatic regulation similar to feeding or sleep, their expression being suppressed after satiation of the mating drive and increased following a period of abstinence [29]. In relatively few species, however, has the potential for co-regulation of mating and shared behaviors in response to changes in internal state been studied in detail. Early evidence for antagonistic co-regulation, or competition, between drives for feeding and mating behaviors came from studies in the sea hare *Aplysia *and related mollusks. As in other species, *Aplysia *exhibit similarities in the motivational regulation of sexual behaviors and homeostatic behaviors such as feeding. For example, failure to satiate drives for either feeding or mating leads to similar increases in arousal and the expression of appetitive behaviors such as swimming [30,31]. However, the expression of feeding and mating behaviors is generally mutually exclusive in these animals [31,32], raising questions as to how animals resolve which behavior to express given their recent history of feeding and reproductive activity. Interestingly, it was found that abstinence from one behavior (by deprivation of food or mates) leads not only to increased expression of that behavior, but also to inhibition of the other behavior [31,32]. This reciprocal regulation indicates that animals resolve conflicts between these two mutually exclusive behaviors by changing their behavioral priorities as a function of internal state. Even in these relatively simple animals, the motivational drives for mating and feeding can also interact in more complex ways. For instance, exposure to reproductive pheromones can promote feeding in *Aplysia *[33,34], indicating that the energy-intensive nature of reproduction can in some circumstances lead to coordinated upregulation of these behaviors. Indeed, a recent study in *Drosophila *has provided evidence for similar phenomena in this species, where a male-specific olfactory circuit promotes sexual behaviors in response to the detection of food odors [35]. However, it is important to note that in neither of these cases are sexual and feeding behaviors expressed simultaneously, indicating that these behaviors nonetheless remain in competition for expression. Together, these findings demonstrate that sex-specific behaviors exhibit motivational regulation similar to that of behaviors aimed at homeostasis, and compete with these behaviors for mutually exclusive expression.

While these behavioral studies in mollusks offer important insight into how drives for feeding and reproductive behaviors interact, relatively little is known about the underlying neural mechanisms. Recent insights into the regulation of feeding and sexual behaviors in other animal models, however, have shown that modulated sensory function can play a central role in regulating behavioral decision-making. Elegant work in *Drosophila *has recently shown that insulin and neuropeptide Y (NPY), conserved hormonal regulators of feeding, modify foraging behavior as a function of feeding status by altering the sensory representations of food-related odorants [36]. Fasting has also been associated with sensory suppression phenomena, wherein responses to noxious mechanical or thermal stimuli are suppressed when the opportunity to feed arises [37]. Work in *C. elegans *and the leech has shown that serotonin in particular has a key role in mediating these changes in behavioral sensitivity as a function of feeding status [38-41], implementing the prioritization of feeding over responses to noxious stimuli in starved animals. Similar mechanisms have also been implicated in behavioral choice in the context of reproductive behaviors. Studies of moths have shown that the responses of neurons in the male antennal lobe (the primary olfactory center in insects) to female sex pheromone are enhanced by serotonin [42], and it has been speculated that modulations of mate-seeking behavior as a function of circadian cycle [43] and satiation of the mating drive [44] may occur through such neuromodulatory mechanisms [43]. Neuromodulators such as insulin, ghrelin, leptin and neuropeptide Y have long been known to act on central circuits in the mammalian hypothalamus in the regulation of feeding and appetite [6], and various monoaminergic and peptidergic transmitter systems have been implicated in regulating sexual motivation [27,45]. These findings from invertebrate systems suggest additional mechanisms by which these molecules could shape the behavior of higher animals, and provide new mechanistic explanations for earlier psychological findings in humans showing that motivation (*i.e.*, satiety versus hunger) can powerfully modulate the visual, olfactory, and gustatory perception of food [46-48]. Indeed, recent findings suggest the machinery for sensory modulation exists in vertebrate species [49,50], including man [48], to regulate behavioral choice.

Together these findings raise the interesting possibility that prioritization of mating and feeding behaviors may be regulated, at least in part, through the modulated function of early sensory pathways. Notably, emerging work in the nematode *C. elegans *directly supports this idea. This species features two sexes: a hermaphrodite and a male. The hermaphrodite is an anatomically female animal, but transiently produces and stores sperm for self-fertilization, and thus is capable of reproducing in isolation from conspecifics. By contrast, the *C. elegans *male is only cross-fertile, and requires an adult hermaphrodite mating partner to reproduce. While isolated larval animals or adult hermaphrodites will typically remain at a food source indefinitely, adult male nematodes placed alone on a patch of food will eventually abandon it. This tendency to leave food can be suppressed by providing a suitable mate, as well as by briefly fasting the male, suggesting that food-leaving reflects a balance between feeding and mate-seeking drives that is prioritized differently between males and hermaphrodites [51].

**Figure 1:**
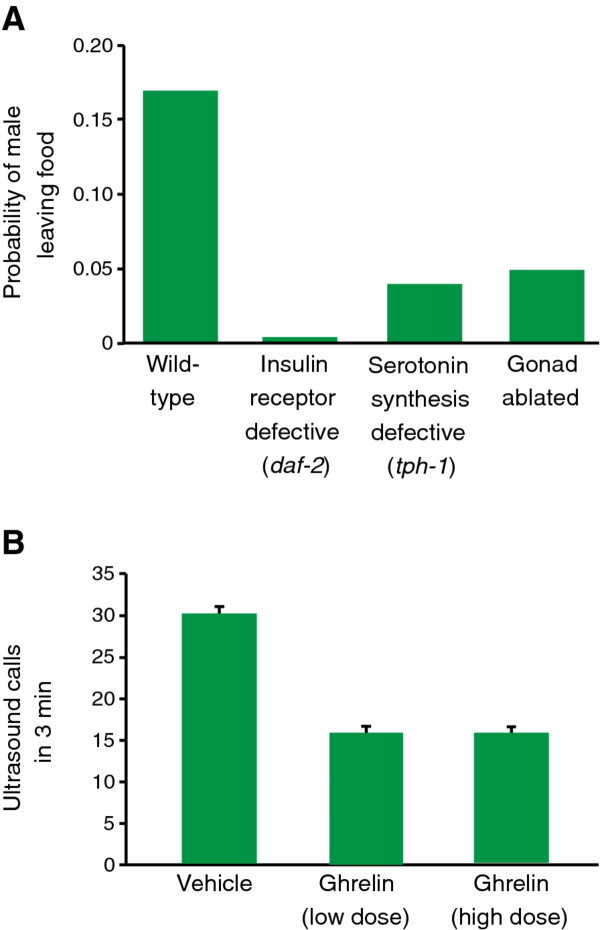
**The expression of mutually-exclusive shared and sex-specific behaviors is decided by interactions of monoamine, peptide, and lipid hormone pathways**. A. Food leaving is a behavior exhibited by *C. elegans *males in the absence of a mating partner. The probability of a male leaving food is regulated by serotonin, insulin, and signals from the gonad (adapted with permission from [51]. Copyright 2004, Society for Neuroscience). B. Hunger may regulate the probability of expressing mating and courtship behaviors in mammals. Ghrelin, a peptide hormone signaling nutritional status, regulates the frequency of ultrasonic courtship calling behavior in mice (adapted with permission from [55]. Copyright 2010, Elsevier Ltd.).

This balance of drives, which may be critical to ensuring male reproductive success in the wild, is established through competing signals. Ablation of either the gonad or of sex-specific sensory organs suppresses the food-leaving drive of adult males, suggesting that signals from the germline as well as male-specific neurons promote an innate propensity toward exploration in this sex [51-53]. Evidence indicates that the gonad-generated signal is a lipid hormone detected by the nuclear hormone receptor DAF-12 [53], while the precise nature of signals from male sensory neurons that regulate leaving remain unclear. Signals that communicate feeding status and food availability are also important in calibrating this balance, as animals lacking insulin or serotonin signaling also suppress exploration [51]. Thus, a balance of chemical signals reflecting sexual status (the gonad and male sense organs) and feeding status (insulin and serotonin) regulates the drive to feed in *C. elegans *(Figure 1A). The mechanisms by which these signals modulate behavior, however, have remained elusive. Recent results indicate that both feeding status, developmental stage, and the sexual identity of shared sensory neurons modulate the expression of a receptor for a food-related odorant, modulating the behavioral response of these animals to food cues (K. Lee *et al.*, manuscript in preparation). Thus, differential sensitivity to food cues could underlie at least part of a mechanism that allows dynamic prioritization of feeding *vs*. exploration in *C. elegans*. How sex, developmental stage, and environment converge on sensory function to modulate behavioral choice in this species remains to be determined. An intriguing possibility is that neuromodulatory or hormonal cues may exert their effects on behavioral decision-making in part through changes in sensory receptor expression.

Importantly, this notion that competitive interactions between hormonal and neuromodulatory signals can mediate sex-specific behavioral prioritization have been echoed in vertebrate studies. Steroid hormones can modulate olfaction in the context of reproductive behaviors in mice, rats, and humans [54], raising the possibility that hormonal regulation of olfaction may also affect feeding behavior in these species. Recent observations also show that ghrelin, a hunger-stimulating peptide released by the gut, is capable of not only promoting food-seeking olfactory behaviors in both rats and humans [48], but also suppresses androgen-regulated mating and aggressive behaviors in mice [55] (Figure 1B). Further, it has been proposed that NPY may also participate in the mutual regulation of feeding and reproductive behaviors in mammals [56]. Thus, a scenario where competition between reproductive and feeding behaviors is mediated by neuromodulatory peptide signals and hormones, as has been proposed for the worm, is likely to be true in mammals, as well. Recent findings from invertebrates suggest that we might look beyond familiar central circuits, such as those in the hypothalamus, to early sensory pathways as potential targets of modulation in sex-specific decision-making.

### Modulation of food-preference in the sexes: Links between reproduction and sensory physiology

**Figure 2:**
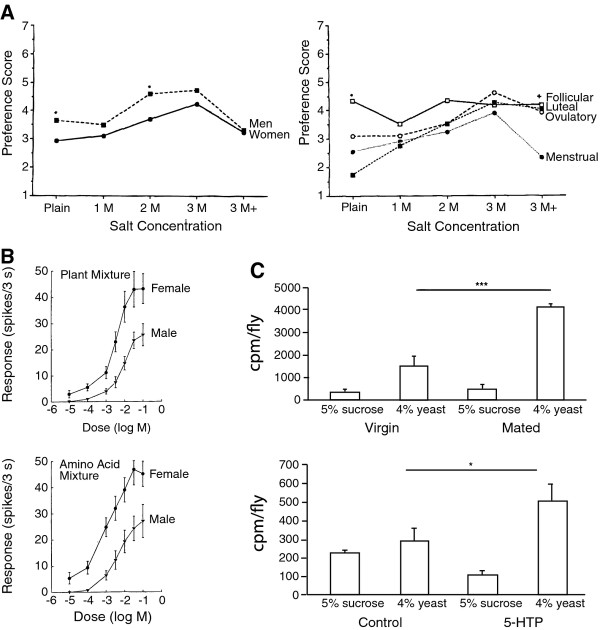
**Sex-specific food-preference decisions are influenced by modulated sensory function**. A. Humans exhibit sex differences in preference for salt, and salt preference in women changes over the menstrual cycle. The mechanisms underlying these different food preferences are unknown (reprinted with permission from [67]. Copyright 1994, Elsevier Ltd.). B. Female fiddler crabs exhibit greater behavioral sensitivity to food stimuli. Sex differences in physiological responses of sensory neurons to food-related stimuli in crabs mirror differences in behavior (reprinted with permission from [71]. Copyright 1995, Springer-Verlag) C. Serotonin is known to regulate carbohydrate consumption in several species. Treating *Drosophila *females with the precursor for serotonin elevates the level of this neurotransmitter, and mimics food-preference changes that occur as a function of reproductive status (Reprinted with permission from [80]. Copyright 2010, Elsevier Ltd.).

The sex of an animal can also influence shared behavior in ways outside the competition between shared and reproductive behaviors. Feeding behaviors are an important example of this phenomenon, where biological sex has been observed to dramatically affect the preference for the taste and smell of specific food items [57], as well as overall food intake [58]. The energetic and nutritional demands of reproduction often differ significantly between the sexes, and thus may play an important role in establishing differences in the kinds of nutrition animals seek. A wide variety of animal species are known to exhibit self-regulated dietary intake, wherein animals will preferentially consume different foods depending on their current nutritional status. The classic work of Curt Richter demonstrated that both rats and humans have the capacity to self regulate the intake of specific nutrients as a function of physiological need, including salts, carbohydrates, and amino acids [59-62]. Further, it has been found in rats and a variety of insect species that females regulate their dietary intake not only as a function of their nutritional status, but also of current reproductive status [18,58,63,64]. In cockroaches, it has been found that this self-regulation of diet can significantly impact lifespan and reproductive fitness [65], indicating the adaptive significance of this ability to modulate feeding behavior. Notably, men and women exhibit significant differences in olfactory discrimination ability [10,66], as well as more specific differences in the gustatory [67], olfactory [66], and visual [68] perceptions related to food. Moreover, gustatory and olfactory perception of food is also altered by the phase of the menstrual cycle and pregnancy in women, suggesting that sensory capacities in humans are also modulated by reproductive status [66,69,70] (Figure 2A).

This similarity in the sex-specific regulation of feeding across diverse species raises the possibility that conserved mechanisms may mediate sex differences in dietary preference. Two general mechanisms have been proposed for mediating the self-regulation of dietary intake: changes in sensory physiology (implicated in psychological phenomena such as sensory-specific satiety [47]) and changes in the feedback received after the animal ingests a specific food (also known as the "malaise hypothesis" [60]). Recent studies in invertebrate models have suggested that sex differences in the regulation of dietary intake may result, at least in part, from changes in the function of shared sensory systems. Early evidence for sex differences in food-detecting chemosensory abilities comes from studies of fiddler crabs, where it was found that increased female behavioral sensitivity to certain food cues is correlated with enhanced sensitivity of gustatory afferents in the claw and legs to these stimuli [71,72] (Figure 2B). Such specializations of non-pheromonal chemosensory circuitry have since been documented in a variety of insect models, with the discovery of sex-specific non-pheromonal taste receptors in *Drosophila *[73] and sex differences in odorant receptor expression and sensitivity to host plant volatiles in the olfactory neurons of several species of moth [74-77]. In *C. elegans*, genetic methods have demonstrated that sex differences in chemosensory preference behavior also rely on the functional modification of shared chemosensory organs [78]. Modified sensory function has also been implicated in mammalian behavioral sex differences, as classical sex differences in salt taste preference in rats have recently been found to be related to differences in sensory physiology of taste receptors [79]. Together, these results suggest that functional modifications of sensory circuitry may underlie sex-specific dietary preference behaviors in a broad array of animal species.

Here again, neuromodulators have been shown to have key roles in imparting sex differences to behavioral decision-making. *Drosophila *adult males and females differ in their dietary preferences [80,81], with females consuming more protein-rich yeast than sucrose. This preference of females for yeast is exaggerated in response to mating, indicating that *Drosophila *regulates dietary preference in response to reproductive status [80,81]. Sex peptide and other components of the male seminal fluid act on the nervous system of the female to promote this dietary switch [81]. Interestingly, modulation of the conserved nutrient-sensing TOR pathway in the nervous system plays an important role in changing dietary preference [80,81], though this modulation appears to occur independent of input from the insulin pathway [81]. Neural TOR signaling in turn modulates nutrient preference at least partly by increasing levels of the neuromodulator serotonin in the brain [80] (Figure 2C). Significantly, serotonin has also been previously implicated in regulating the ratio of protein to carbohydrate consumption in cockroaches [82] and rats [83-86], suggesting that such a mechanism could be conserved in more complex species. Evidence from *Drosophila *thus suggests that modulation of dietary preference by sex and reproductive status involves the action of a conserved nutrient-sensing and neuromodulatory pathways. How exactly these pathways alter neural function to bring about changes in dietary preference behavior remains unclear. It will be interesting to see if these pathways again converge on shared sensory mechanisms to impart sex differences to shared behavior.

### Sex differences in motor function: Manifestations of changes in motivation?

In addition to sensory function, the sex of an animal can also affect shared aspects of motor behavior, such as locomotion, feeding, and respiration. Though sex differences in these aspects of behavior have often been explained as a secondary consequence of morphological disparities [87], recent evidence has pointed to the nervous system as an important driver of sex differences in motor behavior [87,88]. In several cases, these differences can be seen as stemming from sex differences in motivational state or arousal. Switching between motor patterns, as well as the kinematics of motor programs, can both be altered by an animal's internal state. For example, feeding episodes in pond snails are modulated in duration by hunger [89], and competition between the distinct ingestion and egestion motor programs is regulated by hunger and NPY in *Aplysia *[90], ultimately regulating the rate of food intake. In *C. elegans*, feeding status modulates the rate switching between forward and reverse locomotion [91,92], as well as locomotor kinematics [93], resulting in qualitatively different patterns of exploratory behavior. Several studies have suggested that monoamine neurotransmitters signaling feeding status, including dopamine [92], serotonin [92,94], and octopamine [95], regulate these shifts in *C. elegans *motor behavior. As many of the mechanisms that modulate motor behaviors have also been implicated in broader changes in affect and motivational state, this raises the possibility that sex differences in motor behavior may be intimately connected to sex differences in decision-making.

Recent work in invertebrate models suggests that sex differences in motor behavior may indeed be connected to sex-specific regulation of behavioral state, at least in some instances. Bouts of locomotor behavior in adult *Drosophila *have been observed to differ according to an animal's sex, with males exhibiting more consistent, stereotyped locomotor activity compared to females [96,97]. These sex differences in locomotor activity are dependent on insulin and juvenile hormone signaling [98,99]. As these hormones that have been implicated in the internal representation of feeding status in insects [100-102], these observations raise the possibility that sex differences in spontaneous motor activity may reflect a sex difference in appetitive or motivational state in *Drosophila*. It is thought that sexual modification of a small set of approximately ten neurons in the *pars intercerebralis *(PI) determines the sex-specific structure of locomotor bouts. As these neurons project onto cells in the juvenile hormone-synthesizing region, the *corpus allatum*, it is hypothesized that they may directly regulate the secretory activity of these cells. Interestingly, neighboring cells in the PI produce insulin, and the ablation of these cells feminizes the locomotor activity of males [99]. It remains unclear, however, whether insulin signaling itself is sexually regulated, or if it acts in a parallel pathway to promote the expression of locomotor sex differences [99]. Sex differences are also a prominent feature in *C. elegans *locomotion, with higher male locomotor activity being regulated by the sexual modification of shared neural circuits (W.R.M and D.S.P, in preparation). Similar to food-leaving behavior, enhanced locomotor activity may promote mate-finding and male fitness in this species. While increased locomotor activity does not itself cause food-leaving, there may nonetheless be deep similarities in the motivational control of these behaviors that serve to optimize male-specific reproductive fitness. In this sense, sex differences in shared motor behaviors may be linked to broader differences the motivational state of the sexes.

Notably, these studies have an important connection to human behavior, as there is significant sex bias in behavioral disorders such as ADHD where normal regulation of motor activity and arousal are disrupted [103]. Dysregulation of signaling through dopamine and other monoamine transmitters has been implicated in the impulse control, attentional, and emotional disturbances characteristic of this disorder [24,104-106]. The notion that monoamine neurotransmitters may play a causal role in this disorder is supported by an extensive non-human primate literature documenting the importance of dopamine in the regulation of prefrontal cortex and executive function [107]. Indeed, current pharmacological interventions for ADHD, such as methylphenidate (also known as Ritalin) and Adderall (an amphetamine mixture), target these very neuromodulatory systems, increasing the concentrations of dopamine and norepinephrine in the brain. However, much remains unknown as to how these neurotransmitters function in the regulation of motor activity and attention. Further, there is little data to explain why ADHD exhibits such profound sex bias. The links discovered between sexually differentiated locomotor behavior and neuromodulatory signaling in invertebrates are particularly intriguing in this regard. These studies offer the opportunity to uncover important, and perhaps conserved, mechanistic links between neuromodulatory signaling and the motivational regulation motor activity, and understand their sex-specific regulation.

### "Sex-specific" social behaviors can emerge through the modulation of shared circuits

Social behaviors are crucial to the reproductive success of animals. Aggressive behaviors can enable an individual to secure mates and resources crucial to reproduction. Courtship rituals are similarly important in allowing an individual to gauge the fitness of potential mates. When an animal is confronted with a conspecific of the same or opposite sex, a decision-making process is engaged: an optimal response must be selected from a repertoire of social behaviors which includes aggression, courtship behaviors, and affiliative behaviors, amongst others. Typically, the choice of response in such a situation depends heavily on the sex and reproductive status (*e.g.*, sexually mature vs. juvenile) of the individuals involved. For example, adult males in a given species may consistently engage in aggressive interactions with other adult males, but attempt to court or mate with adult females.

The highly sex-typical outcome of this behavioral decision-making process has often led to the characterization of some social behaviors as being "sex-specific." It is important to note, however, that many elements of these behaviors are commonly shared by both sexes, with sex differences emerging instead through the alterations in the frequency, intensity, and context-specificity of behavioral expression. Aggressive behaviors, for example, are commonly observed in both sexes, though particular patterns of aggressive behavior exhibit strong sex-bias [108]. Similar findings have arisen in relation to mounting behaviors in mammalian species. While males often employ these behaviors in the context of mating, females can also exhibit mounting in non-sexual contexts, such as in establishing intrasex dominance relationships [109,110]. In some species, the sex-specificity of certain behaviors can vary considerably with genetic background. Indeed, there are strains of rats in which up to 50% of males will exhibit "female-specific" lordosis and other proceptive behaviors in response to other males [3,111]. This notion that the capacity for some "sex-specific" behaviors is actually shared by the sexes is supported by investigations of the neural mechanisms supporting these behaviors. In *Drosophila*, it has been shown that direct optogenetic stimulation of flight circuitry can elicit male-like wing song behavior in females [112]. Similar findings have arisen in mammals as well. For example, it has been found that ablation of the vomeronasal organ in mice can "unmask" male-typical patterns of sexual and aggressive behaviors in females [113]. Together, these findings challenge the intuitive notion that the production of sex-specific behaviors should require dedicated sex-specific circuitry (codified in the theory that sex hormones act early in vertebrate development to organize sex-specific circuitry that allows one sex to produce sex-typical behaviors [114]). Rather, it appears in some cases that sex-specific regulation of nominally shared circuitry may be crucial to characteristic sex differences in the frequency and context-specificity of behavioral expression.

**Figure 3:**
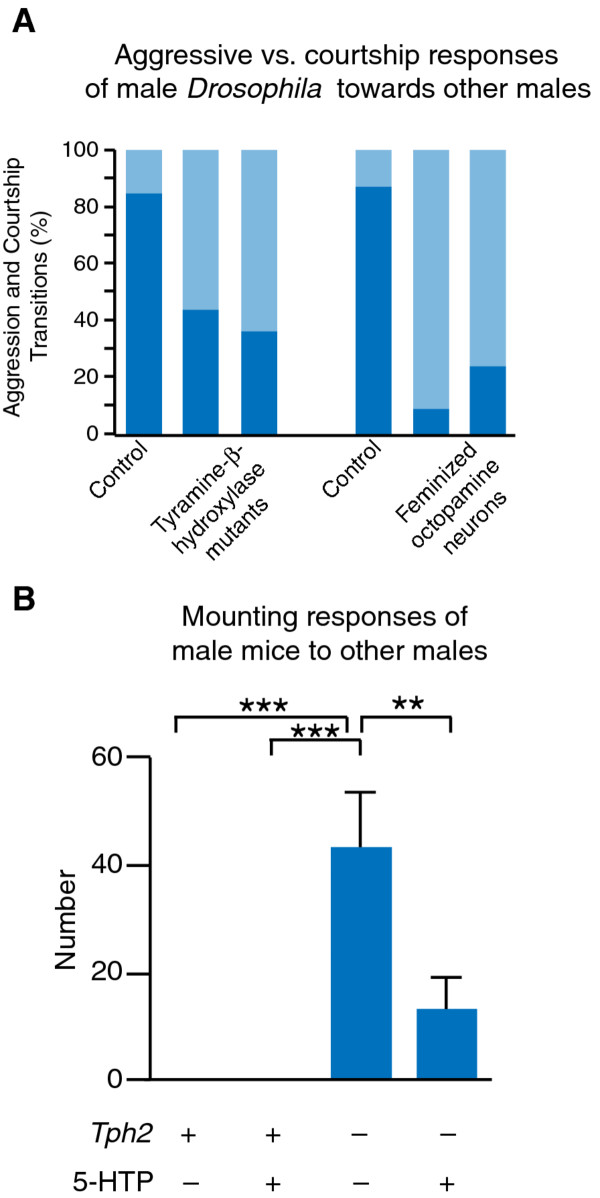
**Sex-specific social decision-making is regulated by monoamine signaling**. A. Male *Drosophila *typically respond with aggression towards other males, though they will sometimes attempt to court them. This decision between aggressive and courtship responses to same-sex conspecifics is regulated by sex-specific octopamine signaling. Both loss of the ability to synthesize octopamine, and genetic feminization of octopaminergic neurons, results in males that court other males with increased frequency (adapted with permission from [116]. Copyright 2007, The National Academy of Sciences of the USA). B. Male mice respond to other males with aggression much more frequently than courtship or mating behaviors. This decision to respond to other males with aggression, rather than mating, is regulated by serotonin signaling. *Tph2 *mutant male mice defective in serotonin synthesis have dramatically increased frequency of mating behaviors directed towards other males. This behavior can be partially rescued by treating animals with the serotonin precursor 5-HTP, bypassing the requirement for the *Tph2 *gene in serotonin synthesis (adapted with permission from [121]. Copyright 2011, Macmillan Publishers Ltd.).

How does an animal's sex modify the behavioral decision-making involved in conspecific interactions, leading to differences in the frequency and contexts of behavioral expression? Investigations of courtship behavior in *Drosophila *have indicated that neuromodulatory pathways play a crucial role in regulating behavioral decision-making during social interactions. Increased activity in circuits releasing octopamine (a transmitter thought to have functions similar to vertebrate norepinephrine) can promote male-male courtship in *Drosophila *under circumstances that would normally evoke aggression [115]. Strikingly, when the same sets of cells are genetically feminized, male-male courtship is also enhanced [115,116], suggesting that sexual modification of octopamine signaling may indeed be involved in establishing mate preference in *Drosophila *(Figure 3A). Additional neuromodulatory pathways, including dopamine and the hormone ecdysone, have also been implicated in the regulation of *Drosophila *mate preference, though to date none of these molecules have been linked to the mechanisms of sexual differentiation [117-119]. These findings in fruit flies clearly show that sexual modification of shared neuromodulatory circuits can influence behavioral decision-making in social interactions. Important parallels of this work in *Drosophila *have recently been discovered in the context of conspecific interactions in mice. Until very recently, the normal aggressive response of adult male mice to one other was only known to require a functional vomeronasal organ, presumably necessary for proper sex discrimination based on pheromonal cues [120]. However, a recent study has shown that monoamine signaling is involved in determining the response of male mice to other males. Adult male mice defective in serotonin signaling lose their normal mating preference for females over males, and attempt to mate with male mice rather than engage in agonistic behaviors [121] (Figure 3B). While a different monoamine transmitter system was investigated in this study, this finding reinforces the idea that there may be a conserved role for monoamine systems in regulating the sex-specific character of social behaviors, which may be elucidated through further studies on invertebrate models.

## Conclusions

**Figure 4:**
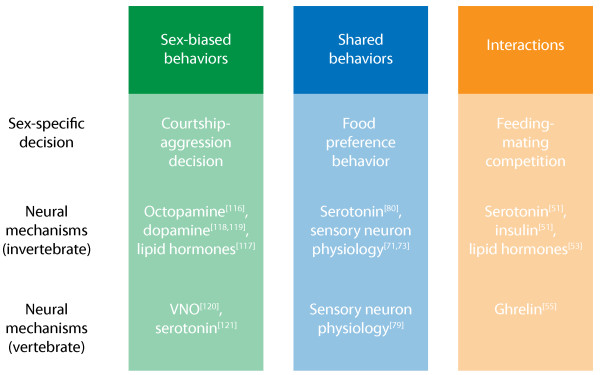
**Sex differences in behavioral decision-making and the modulation of shared circuits**. Sex differences in three different classes of behavioral decisions are shown, together with shared neuromodulatory mechanisms that affect sex-specific decision-making in both invertebrate and vertebrate systems. In some cases, such as octopamine signaling in *Drosophila*, these systems are known to undergo sex-specific modification.

The evidence that modulated function of shared circuitry plays a central role in establishing sex differences in behavioral decision-making is strong and arises from diverse sources. Studies on the sex-specific interaction of feeding and mating drives has shown that competition between these behaviors is mediated, at least in part, through modulation in the sensitivity of shared olfactory structures. Competition between these drives further involves the complex interaction of hormonal, peptide, and monoamine signaling mechanisms, though the connection between these conserved neuromodulatory mechanisms and regulated sensory function is in many cases unclear. Sex-specific regulation of sensory function has also been implicated in mediating sex-specific dietary preference in a number of species. Again, monoaminergic signaling mechanisms have been implicated in regulating these behavioral sex differences, though the specific effects on sensory processing remain to be elucidated. Neuromodulatory systems, including insulin and hormonal signaling, have further been implicated in the regulation of sex differences in motor activity, suggesting that such sex differences may be linked to broader differences in motivational state of males and females. Finally, we have cited abundant evidence that the capacity for sex-specific behavior is in many cases present in both sexes. Studies of *Drosophila *courtship and rodent mating behavior have illustrated the idea that monoamine signaling can play an essential role in determining the sex-specific nature of social interactions. Together, this evidence indicates that neuromodulatory systems play a central role in implementing sex differences in shared circuit function and behavior (Figure 4).

A crucial open question is the degree to which the expression of behavioral sex differences simply *depends *upon neuromodulation, as opposed to the notion that these differences *emerge from *sex differences in neuromodulatory signaling. Several cases indicate that the latter applies in at least some situations. A prime example would be the demonstration that male decision to fight, rather than court, other males in *Drosophila *is regulated by the action of the sexual differentiation factor *fruitless *in octopamine neurons [116], indicating that regulated octopaminergic transmission determines the sex-typical outcome of this decision-making process. Other cases can be more ambiguous. For example, it is known that sexual differentiation of sensory neurons is critical to regulating sex-typical olfactory preference decisions in *C. elegans *[78]. It remains unclear, however, whether sex-specific sensory neuron function results from sex-specific neuromodulation. It is easy to imagine that changes in the expression of monoamine or peptide receptors, as occurs in the sensory neurons of fasted *Drosophila *[36], could play an important role in this process. However, it remains to be seen if this is indeed the case. Examples such as this highlight the fact that the role of shared circuitry in mediating sex-biased or sex-specific behaviors is often poorly defined. Indeed, the extent to which anatomically similar circuitry is modified at the level of fine-scale connectivity, or even at the molecular level (*e.g.*, differential expression of neurotransmitter receptors), is poorly defined in any system. As such modifications may be crucial to establishing adaptive sex differences in animal behavior, a concerted research effort to identify the full extent of these sex differences in animal nervous systems is warranted.

An alternative to the model that sex differences in neuromodulation mediate behavioral differences is that sexual differentiation of shared circuitry acts in parallel to these neuromodulatory mechanisms. As sexual differentiation occurs in large part through regulated gene expression, there are numerous ways in which this process could alter neural function outside of direct effects on neuromodulator signaling. For example, a shared circuit may require neuromodulatory input for its normal function, but be sexually differentiated in its intrinsic excitability or synaptic partner choice, leaving the neuromodulatory input essentially unchanged. A particularly intriguing possibility in this vein is that sex-specific hormones may themselves directly participate in neuromodulation through mechanisms acting in parallel to conventional monoamine and neuropeptide modulators. Accumulating evidence indicates that sex hormones in vertebrates have diverse actions on neural circuitry outside of regulating gene expression, including activation of ion conductances [122]. Indeed, it has been known for some time that molecules such as 17-β-estradiol can have rapid effects on the reproductive behaviors of rodents, modulating the motivation and performance of these behaviors on a minute-by-minute basis [123]. Interactions of estradiol with neurotransmitter receptors, such as GABA, NMDA, and dopamine receptors, have been implicated in mediating the rapid regulation of lordosis in female rats [123]. Further, it has been suggested that monoamine neurotransmitters such as dopamine can modulate sexual receptivity in female rats by binding to steroid receptors themselves [124], reinforcing the idea that hormonal and monoamine systems interact extensively in the regulation of behavior. Sex hormones may thus act as neuromodulators themselves, shaping the activity of neural networks on a relatively rapid time scale. Evidence from invertebrate models suggests that these roles for hormonal regulators participate in the regulation of sex-specific behavior may be broadly conserved across animal species. In *Drosophila*, ecdysone has been implicated as an important regulator of sexual preference in males [117], and juvenile hormone has been shown to regulate sex differences in locomotor activity [99]. In *C. elegans*, the nuclear hormone receptor DAF-12 is capable of "activating" male mate-searching behavior [53]. Thus, lipid-derived hormones play an important (and perhaps conserved) role in the rapid sex-specific modulation of behavior in a broad array of animal species. Invertebrate models provide an important opportunity to gain insight into the mechanisms underlying the ability of these molecules to orchestrate behavioral change.

Significantly, the modulatory pathways that mediate sexual regulation of behavior may also give rise to sex bias in the susceptibility to neurological and mental health disorders in humans. Altered neuromodulatory signaling has been implicated in a wide variety of mental health disorders, including schizophrenia, depression, ADHD, and autism [21-24], disorders that also exhibit significant sex-bias in their incidence [19]. Notably, the behavioral capacities affected by these disorders are not sex-specific, raising the possibility that subtle sex differences in the organization or function of neural structures mediating shared behaviors may underlie this sex bias. A number of the invertebrate studies cited above have indicated that sex differences in neuromodulation can alter behavioral processes affected by these disorders, such as sensory perception, motor activity and arousal, and social interactions. While these organisms cannot directly recapitulate the complexity of human behavioral disorders, numerous parallels between sex differences in these animals suggest that core mechanisms mediating behavioral sex differences are conserved. Supporting this idea, a number of these findings have been extended to explain sex differences in the behavior of vertebrate species, including the roles of monoamine transmitters in regulating sex-specific social behaviors, of modulated sensory function in mediating sex differences dietary preference, and of peptide neurotransmitters in regulating the competition between shared and sex-specific behaviors.

Together, these findings suggest that investigating the mechanistic underpinnings of behavioral sex differences in invertebrates can shed important light on the sources of sex bias in human neurological and behavioral disorders, particularly those affecting shared behaviors. However, significant differences exist in sex-determination mechanisms of even closely related species [125], raising questions about whether mechanisms regulating sex-specific behavior may be conserved. For invertebrate and vertebrate species where sex determination is well-understood, including *C. elegans, Drosophila melanogaster*, and *Mus musculus*, there appears to be little conservation in many of the molecular and genetic mechanisms involved [126]. An important exception to this general rule are a family of genes, the DM-domain transcription factors, which have been implicated in the sex-specific development of a variety of invertebrate and vertebrate species [126-128] (including humans [129-131]). Indeed, in both *Drosophila *and *C. elegans*, DM genes play important roles in the sexual differentiation of neural circuitry and behavior [132-135], hinting that they may have similar roles in vertebrates as well.

Thus an appealing model, based initially in theory [136] and subsequently substantiated by the discovery of the conservation of DM genes [127], is that upstream mechanisms of sex determination are highly divergent but downstream effectors of sexual differentiation can be conserved [126,136,137]. In this sense, the distinct chromosome-counting mechanisms of invertebrates and the *Sry- *and hormone-driven pathways of mammals may converge directly or indirectly on conserved factors, including DM genes, to bring about specific differences in neural development and circuit modulation. For the reasons discussed above, neuromodulatory genes—*e.g.*, neuropeptides or their receptors—are particularly attractive candidate targets for regulation by cell-autonomous and hormone-mediated sexual differentiation pathways. Though it remains to be seen whether the neuromodulatory mechanisms that help implement sex differences in behavioral decision-making reflect evolutionary conservation or convergence, investigating the mechanisms that establish sex differences in the behavioral decision-making of invertebrates will enrich our understanding of the astonishing flexibility and adaptability of nervous systems. Even in the absence of direct conservation, insights from invertebrates may yet direct us towards general principles of organization and function that underlie these properties.

## Competing interests

The authors declare that they have no competing interests.

## Authors' contributions

WRM and DSP discussed the ideas treated herein and drafted an outline of the manuscript. WRM wrote the text, which DSP revised and edited. All authors read and approved the final manuscript.
